# Chemoinformatics Strategies for Leishmaniasis Drug Discovery

**DOI:** 10.3389/fphar.2018.01278

**Published:** 2018-11-01

**Authors:** Leonardo L. G. Ferreira, Adriano D. Andricopulo

**Affiliations:** Laboratory of Medicinal and Computational Chemistry, Center for Research and Innovation in Biodiversity and Drug Discovery, São Carlos Institute of Physics, University of São Paulo, São Carlos, Brazil

**Keywords:** medicinal chemistry, ligand-based drug design, structure-based drug design, neglected tropical diseases, molecular modeling, leishmania

## Abstract

Leishmaniasis is a fatal neglected tropical disease (NTD) that is caused by more than 20 species of *Leishmania* parasites. The disease kills approximately 20,000 people each year and more than 1 billion are susceptible to infection. Although counting on a few compounds, the therapeutic arsenal faces some drawbacks such as drug resistance, toxicity issues, high treatment costs, and accessibility problems, which highlight the need for novel treatment options. Worldwide efforts have been made to that aim and, as well as in other therapeutic areas, chemoinformatics have contributed significantly to leishmaniasis drug discovery. Breakthrough advances in the comprehension of the parasites’ molecular biology have enabled the design of high-affinity ligands for a number of macromolecular targets. In addition, the use of chemoinformatics has allowed highly accurate predictions of biological activity and physicochemical and pharmacokinetics properties of novel antileishmanial compounds. This review puts into perspective the current context of leishmaniasis drug discovery and focuses on the use of chemoinformatics to develop better therapies for this life-threatening condition.

## Current Panorama of Leishmaniasis

Leishmaniasis is a neglected tropical disease (NTD) that causes approximately 20,000 deaths each year. Nearly 300,000 new cases of the disease are registered annually, and over 1 billion people are exposed to the risk of infection^1^. The disease is caused by more than 20 species of *Leishmania* protozoan parasites that are transmitted to humans through the bites of female *Phlebotomus* and *Lutzomyia* sandflies. Leishmaniasis occurs in 98 tropical and subtropical countries encompassing the Mediterranean Basin, South-East Asia, Afro-Eurasia, East Africa, and the Americas. People who are exposed to adverse socioeconomic circumstances, malnutrition, poor housing, and unsanitary conditions are the main target of leishmaniasis (Hailu et al., 2016).

Although leishmaniasis is a curable condition, treatment depends on a variety of factors, including geographic region, clinical form of the disease and parasite species. The available chemotherapy consists of drugs that cause serious side effects, such as renal, pancreatic and hepatic toxicity, teratogenicity, and cardiac and gastrointestinal problems (Copeland and Aronson, 2015). The need for hospitalization, long-term and costly treatment, and drug resistance are additional drawbacks. To this list, one may add the difficulties in implementing the widespread use of the 2014-approved drug miltefosine due to problems of affordability and limited availability and accessibility (Sunyoto et al., 2018). Another current concern in endemic regions is the contingent of patients with leishmaniasis who are coinfected with the HIV virus. Lower cure rates are achieved in these patients because both pathogens attack the immune system. Furthermore, this group is more vulnerable to the drug-associated adverse effects, which contribute to higher death rates (Abongomera et al., 2018). These drawbacks have driven the creation of robust worldwide efforts to pursue novel therapeutic options. This article provides a perspective on these efforts, focusing on recent advances that involve the use of chemoinformatics.

## From Trial-And-Error to Knowledge-Based Drug Design

Similar to most early NTD-focused research programs, drug discovery for leishmaniasis relied on trial-and-error strategies that were based solely on phenotypic screenings. This paradigm reflected the lack of a reasonable understanding of the molecular aspects of the *Leishmania* biology and the cellular processes involved in parasite–host interaction (Gilbert, 2013). This setting began to change when the outstanding findings from genome projects in the mid-2000s started to open an array of new opportunities in leishmaniasis drug discovery (Reguera et al., 2014). Simultaneously, novel collaborative networks were settled, incorporating pharmaceutical companies, and not-for-profit organizations, which, along with research and academic institutions, have brought previously unavailable technological and scientific developments to the field (Preston and Gasser, 2018). Since then, genomics, proteomics, and structural biology data have been made available via open-access NTD-focused databases, which have been essential to the use of chemoinformatics in leishmaniasis research. The Sanger Institute’s GeneDB, for example, organizes the data of several *Leishmania* species and is a useful tool for searching particular gene sequences and investigating gene similarity and function (Logan-Klumpler et al., 2012). Another important virtual platform, the WHO’s TDR Targets Database, is a chemogenomics resource that is focused on NTDs and connects information from diverse protein and small-molecule libraries (Magariños et al., 2012). In doing so, the TDR Targets Database algorithm generates privileged combinations of molecular targets and compounds to be considered for experimental studies. To this list, one may add LmSmdB, which is a database that simulates metabolic networks (Patel et al., 2016), and LeishMicrosatDB, which is a search engine for microsatellite sequences in *Leishmania* genomes (Dikhit et al., 2014). Resulting from these advances, more than 340 protein structures from *Leishmania* spp. are currently registered in the Protein Data Bank (PDB) (Berman et al., 2000). These data have been key to understanding the parasite’s molecular machinery and interspecies variability, which are fundamental aspects to developing broad-spectrum drugs.

Taking advantage of this progress, researchers have increasingly engaged in research and development (R&D) organizational models that are characterized by well-structured worldwide collaboration networks, which are referred to as public-private-partnerships (PPPs) (Preston and Gasser, 2018). These initiatives have been pivotal to enhancing the research infrastructure of NTDs by providing state-of-the-art facilities and technologies, high-quality compound libraries for screening and highly qualified human resources. One noteworthy example is the Drugs for Neglected Diseases Initiative’s (DND*i*) Lead Optimization Latin America (LOLA) consortium, which focuses on preclinical *in vitro* and *in vivo* efficacy, safety and pharmacokinetics assessment^2^. Experimental evaluation is routinely followed by chemoinformatics studies to identify structure-activity and structure-property relationships that guide the design of optimized compounds. The value of this type of initiative has been demonstrated by the successful development of several candidates that are currently undergoing advanced preclinical trials for leishmaniasis^3^.

## Structure- and Ligand-Based Strategies in Leishmaniasis Drug Discovery

Technologies such as combinatorial chemistry and high-throughput screening (HTS) have enabled tests on large compound libraries that encompass a significant chemical diversity in short time scales (Folmer, 2016; Liu et al., 2017). Although these highly impactful approaches have enhanced the potential of the pharmaceutical industry to deliver better drugs in all therapeutic areas, they contributed to scale up the complexity of drug R&D. In this context, in which the outstanding demands for innovation are constantly challenged by significant attrition rates, the industry has put intensive effort into the integration of computational tools into the research pipeline (Rognan, 2017). Being cost-effective mainly in the early stages of discovery, this R&D setting is especially suited to clinical conditions, such as leishmaniasis, which have limited resources compared with mainstream therapeutic areas. Hence, given the ability of chemoinformatics to rapidly estimate ligand-receptor interactions and a number of physicochemical and pharmacokinetics properties, this approach has steadily grown as a key component of drug R&D (Ponder et al., 2014; Macalino et al., 2015).

**FIGURE 1:**
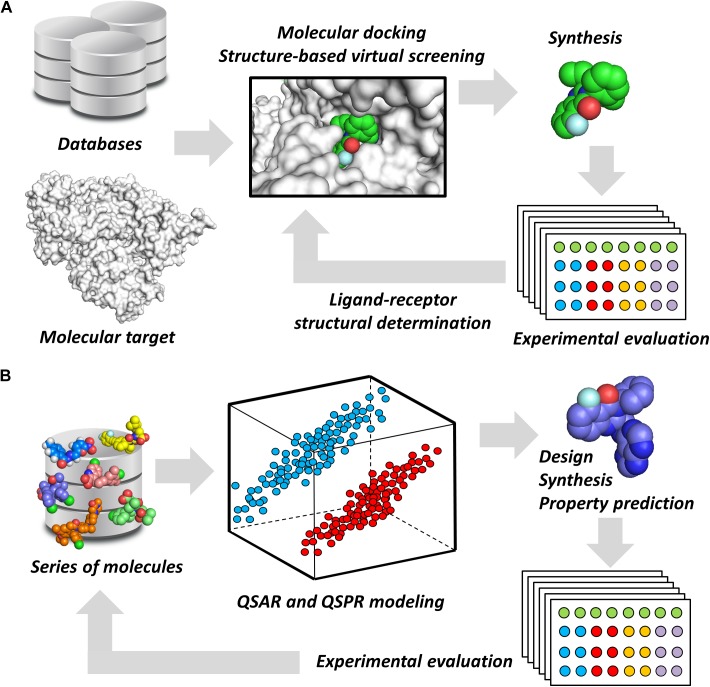
Chemoinformatics strategies. **(A)** SBDD approaches using virtual screening and molecular docking. These methods are useful for revealing phenomena associated with intermolecular interactions and for improving parameters, such as ligand-receptor affinity. Active molecules can have their binding mode experimentally determined by techniques such as X-ray crystallography. **(B)** LBDD and the development of QSARs and QSPRs. These are broadly used for the design of novel compounds and for the prediction of pharmacodynamics and pharmacokinetics properties. The experimental data gathered from newly designed compounds can be added to the dataset to generate enriched models.

Notwithstanding their broad diversity, chemoinformatics tools are generally classified into structure- and ligand-based drug design (SBDD and LBDD, respectively) approaches. SBDD methods consist of the use of the 3D coordinates of molecular targets to investigate and optimize ligand-receptor interactions (van Montfort and Workman, 2017). SBDD programs have revealed the 3D architecture of a variety drug targets, mainly by the use of techniques such as X-ray crystallography. By uncovering binding site attributes, such as shape and electronic distribution, SBDD efforts have been able to deliver ligands with accurately designed properties to achieve high-affinity interactions with their targets (Ferreira et al., 2015). This process is generally assisted by methods such as molecular docking and structure-based virtual screening (SBVS), whereby potential ligands can be evaluated as to their binding mode and energetics (Figure 1A). By examining these data along with experimental results, structure-activity relationships (SAR) can be derived and then used to optimize ligand-receptor affinity and other properties (dos Santos et al., 2018).

Some promising macromolecular targets have been investigated in leishmaniasis drug discovery. The most relevant are topoisomerases and proteases (mainly cysteine-proteases) (Ansari et al., 2017). Other important targets are tubulin, proteins of the folate metabolic route, kinases, phosphodiesterases, and enzymes that are involved in the trypanothione and purine salvage pathways (Ansari et al., 2017). Ligands belonging to a broad variety of chemical classes have been identified for these targets, providing high-quality data for drug design.

Ligand-based drug design studies can be performed without the receptor 3D structure. Instead, they require information on the structure, activity, and molecular properties of small molecules (Chen, 2013). These data are used to construct chemometric models that correlate molecular properties (molecular descriptors) with pharmacodynamics and pharmacokinetics parameters (target properties). In doing so, quantitative structure-activity and structure-property relationships (QSAR and QSPR, respectively) can be derived to identify molecular descriptors that are directly associated with the target property (Yousefinejad and Hemmateenejad, 2015). By providing this type of information, these models are useful for evaluating the target property and guiding the design of new compounds that have improved profiles (Figure 1B). Today, many free-access and commercial software programs that include well-validated QSAR and QSPR models are available for predicting a number of properties. They vary from online platforms that are very straightforward to use to packages that require local license installation.

The use of SBDD and LBDD methods in leishmaniasis drug discovery is an encouraging strategy that has advanced alongside the progress made in the NTD field (Njogu et al., 2016). Chemoinformatics studies have incorporated different SBDD workflows that focus on established and newly discovered molecular targets. On the other hand, the use of QSAR and QSPR models for predicting key pharmacodynamics and pharmacokinetics properties has also been noteworthy. The manipulation of this information, including genomics, metabolomics, structural, and small-molecule data, has been particularly useful for running metabolic network predictions for prospecting novel molecular targets and promising compounds and for proposing likely mechanisms of action. The next sections bring a perspective on a few recent cases using chemoinformatics, focusing on their contribution to the progress of leishmaniasis drug R&D.

### Structure-Based Studies

**FIGURE 2:**
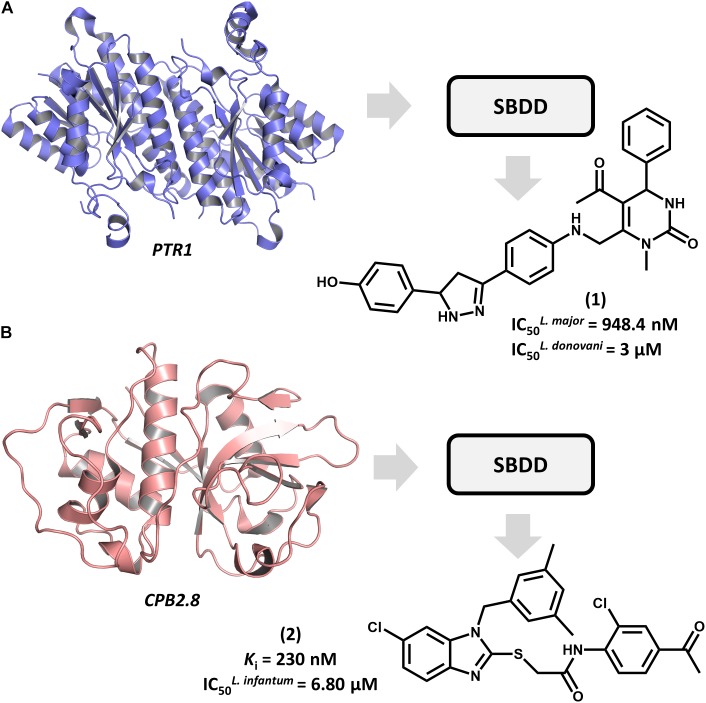
SBDD in leishmaniasis drug discovery. **(A)** An SBDD approach using molecular docking on pteridine reductase 1 (PTR1) that led to the discovery of dihydropyrimidine **1** as a novel antileishmanial agent. **(B)** The design of the *L. infantum* cysteine-protease type 2 (CPB2.8) inhibitor **2** having antileishmanial activity.

Structure-based drug design efforts have prominently contributed to uncovering novel ligands for both well-established and newly discovered drug targets in *Leishmania* spp. One example is pteridine reductase 1 (PTR1), which is an enzyme involved in the pteridine salvage pathway and folate metabolism and a validated target in leishmaniasis drug discovery (Ong et al., 2011). This enzyme was explored in a study that reported on an SBDD strategy for designing novel inhibitors that combine the features of dihydropyrimidine and chalcone derivatives (Rashid et al., 2016). By using the crystallographic structure of *L. major* PTR1, the authors proposed a series of analogs to achieve high-affinity interactions with the catalytic site of the enzyme. Molecular docking-guided structural modifications on the dihydropyrimidine and chalcone moieties and a reduction in the number of rotatable bonds led to the most active compounds against *L. major*. For example, compound **1** proved to be highly active against both *L. major* and *L. donovani* promastigotes, exhibiting a half-maximum inhibition concentration (IC_50_) of 948 nM and 3 μM, respectively (Figure 2A). The predicted ligand-receptor binding energies were consistent with the *in vitro* antileishmanial activity values. These results demonstrate the suitability of these substituted dihydropyrimidines to be further investigated as potential agents against both visceral and cutaneous leishmaniasis.

Among *Leishmania* cysteine proteases, type B enzymes (CPB) have been recognized as key virulence factors whose activity is essential for parasite survival and the invasion of host cells (Casgrain et al., 2016). Within this group, the cathepsin-L-like endopeptidase CPB2.8 has emerged as a promising drug target in leishmaniasis. An article by De Luca et al. (2018) reported the discovery of a series of substituted benzimidazole derivatives that feature nanomolar affinity for *L. mexicana* CPB2.8 (*K*_i_ values ranging from 150 to 690 nM). A few analogs displayed interesting activity on *L. infantum* intracellular amastigotes, with the most potent one (**2**) yielding an IC_50_ of 6.8 μM (Figure 2B). Molecular docking studies were run to examine the binding mode of the compounds within the catalytic site of CPB2.8 and to rationalize the enzyme kinetics data. The administration, distribution, metabolism, excretion and toxicity (ADMET) were predicted to evaluate the drug-likeness of the series and hence, its suitability for further development. Compound **2** demonstrated a good bioavailability profile, which, along with the biochemical and biological results, rendered it a good candidate for future drug design efforts.

**FIGURE 3:**
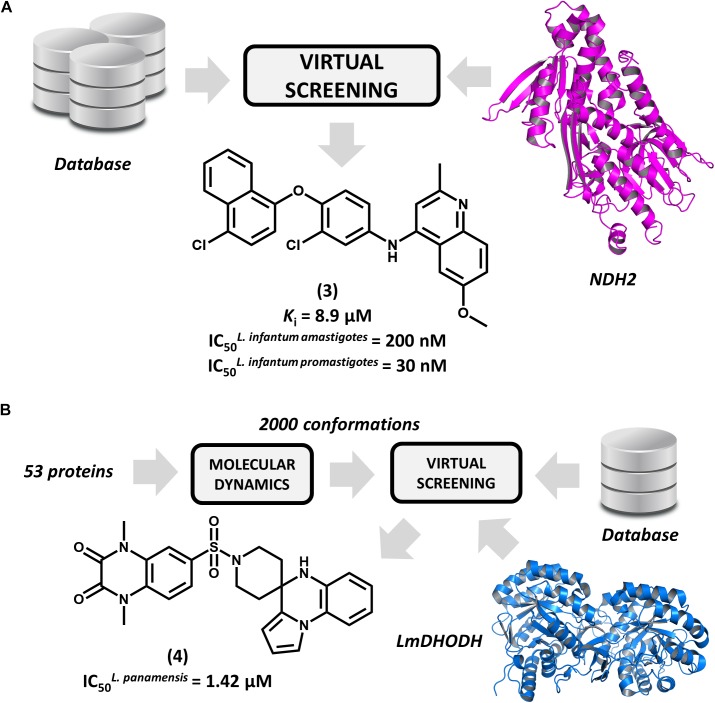
Structure-based drug design (SBDD) strategies using virtual screening and molecular dynamics. **(A)** An SBDD workflow targeting type 2 NADH dehydrogenase (NDH2) resulting in the identification of compound **3**, a remarkably potent antileishmanial agent. **(B)** An SBDD strategy targeting diverse *Leishmania* proteins that led to the discovery of **4**, a novel compound having promising antileishmanial activity.

Type 2 NADH dehydrogenase (NDH2), a mitochondrial enzyme that catalyzes the electron transfer from NADH to ubiquinone, is an emerging drug target in leishmaniasis drug discovery (Marreiros et al., 2017). By constructing a homology model of the enzyme, Stevanović et al. (2018) conducted a pharmacophore-based virtual screening to find novel *L. infantum* NDH2 inhibitors. A group of 23 virtual hits were selected and screened against the recombinant enzyme and subsequently tested for their activity on *L. infantum* whole cells. Out of this set, a 6-methoxy-quinalidine derivative (**3**, Figure 3A) proved to be the best NDH2 inhibitor (*K*_i_ = 8.9 μM). In addition, this compound exhibited nanomolar activity against both *L. infantum* axenic amastigotes (IC_50_ = 200 nM) and promastigotes (IC_50_ = 30 nM). These remarkable results make this novel quinalidine derivative a promising starting point for molecular optimization and *in vivo* studies for visceral leishmaniasis.

Ochoa et al. (2016) reported the use of the IBM World Community Grid to run an SBVS campaign on 53 different *Leishmania* proteins. First, molecular dynamics simulations were performed for this entire set, and then, distinct conformational states of each structure were selected for the SBVS effort. Approximately 2,000 conformations were selected and used to screen a database of 600,000 drug-like compounds, resulting in 1 billion protein-ligand complexes. A group of four proteins were observed engaging in high–affinity interactions with the database compounds, and the most favorable binding energy occurred in *L. major* dihydroorotate dehydrogenase (*Lm*DHODH). This enzyme catalyzes the oxidation of dihydroorotate, a key reaction in the pyrimidine synthesis pathway (Cordeiro et al., 2012). Ten top-scoring *Lm*DHODH inhibitors were selected and evaluated for their *in vitro* antileishmanial activity. Four molecules were active against *L. panamensis* intracellular amastigotes, with the most active one (**4**, Figure 3B) yielding a half maximal effective concentration (EC_50_) of 1.42 μM, which is a value that is comparable to that of the reference drug amphotericin B. Furthermore, this compound showed no toxicity in human macrophages. This compound is a promising candidate for further development, and future investigations are expected to assess its efficacy in reducing *in vivo* parasite burden.

**FIGURE 4:**
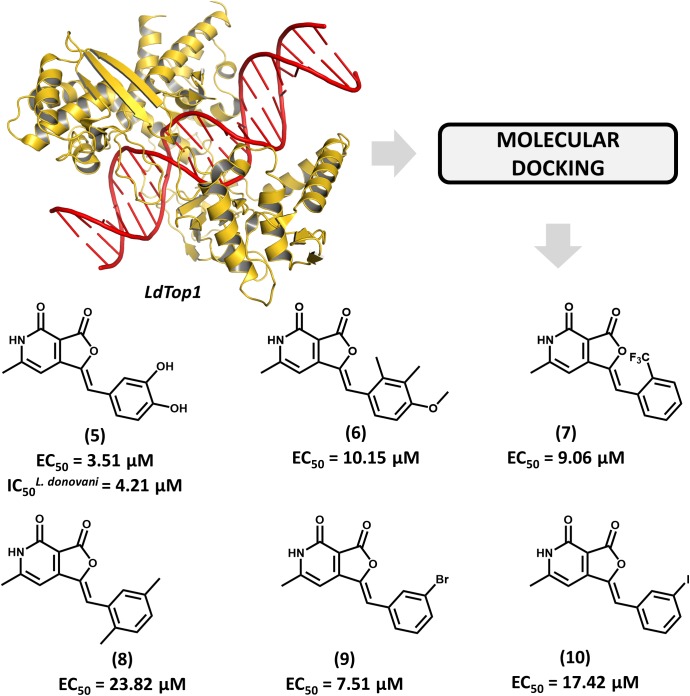
Structure-based drug design approach to the discovery of a series of *L. donovani* topoisomerase 1 (*Ld*Top1) inhibitors. The strategy employing molecular docking led to the identification of compound **5** which shows suitable *in vitro* antiparasitic activity.

The enzyme topoisomerase 1 from *L. donovani* (*Ld*Top1) was selected as the molecular target in an SBDD study by Mamidala and coworkers (Mamidala et al., 2016). The enzyme catalyzes single-strand breaks in DNA, which enables the topological changes that are required during fundamental cellular processes such as gene replication and transcription (Pommier et al., 2016). The authors reported the discovery of a series of *Ld*Top1 inhibitors by using scaffold hopping and bioisosteric manipulations. The structure of known Top1 inhibitors such as camptothecin and edotecarin were used as the starting points for the molecular design. The outline of the compounds was guided by molecular docking runs using the X-ray structures of *Ld*Top1 and the human ortholog. Six compounds showed selective activity against *Ld*Top1 over the human enzyme, yielding EC_50_ values from 1 to 30 μM (**5–10**, Figure 4). The best inhibitor (**5**, EC_50_ = 3.51 μM) exhibited interesting biological activity against *L. donovani* promastigotes (IC_50_ = 4.21 μM) and no toxicity against mammalian cells. The structure of the ternary complex **5**-*Ld*Top1-DNA, which was predicted by molecular docking, revealed key structural features to the design of novel analogs. Considering the suitable antileishmanial activity and the lack of cytotoxicity, further studies on compound **5** would be useful for assessing other aspects, such as its pharmacokinetics profile.

**FIGURE 5:**
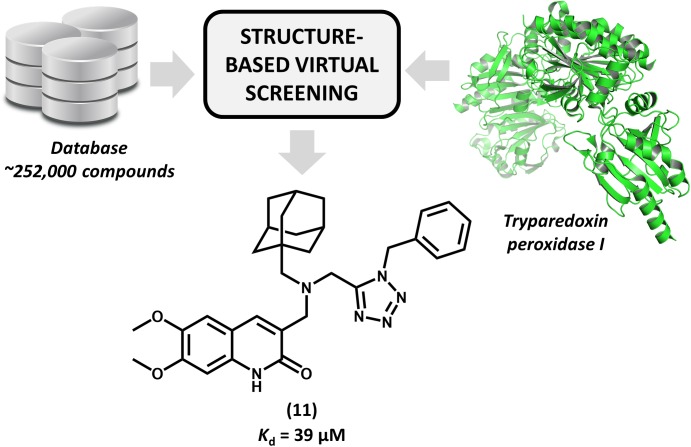
Structure-based virtual screening that resulted in the first report of a series of non-covalent *L. major* tryparedoxin peroxidase I inhibitors. The molecular docking approach led to the identification of aliphatic adamantyl derivative **11** which shows suitable activity against the enzyme.

Brindisi et al. (2015) reported for the first time the discovery of non-covalent tryparedoxin peroxidase inhibitors. Tryparedoxin peroxidase has been considered as a molecular target in SBDD studies since it reduces hydroperoxides produced by infected macrophages. This mechanism of detoxification is particularly attractive for drug design since it is unique to the parasite and essential for its survival (Fiorillo et al., 2012). By using the X-ray structure of *Leishmania major* tryparedoxin peroxidase I (*Lm*TXNPx), the authors run a molecular docking effort and selected a set of hits for experimental profiling. The docking conformations were used for the design of a series of N,N-disubstituted 3-aminomethyl quinolones and some of them displayed activity against LmTXNPx. Forming a number of hydrogen bonds and hydrophobic contacts with the enzyme, the most potent compound (**11**, Figure 5), which has a bulky aliphatic adamantyl system, showed activity in the micromolar range (*K*_d_ = 39 μM). Calculation of physicochemical parameters demonstrated the drug-likeness of the designed series. In view of the activity and the drug-like properties of quinolone derivative **11**, this compound represents a suitable starting point for further studies aiming the development of novel drug candidates against leishmaniasis.

### Ligand-Based Studies

**FIGURE 6:**
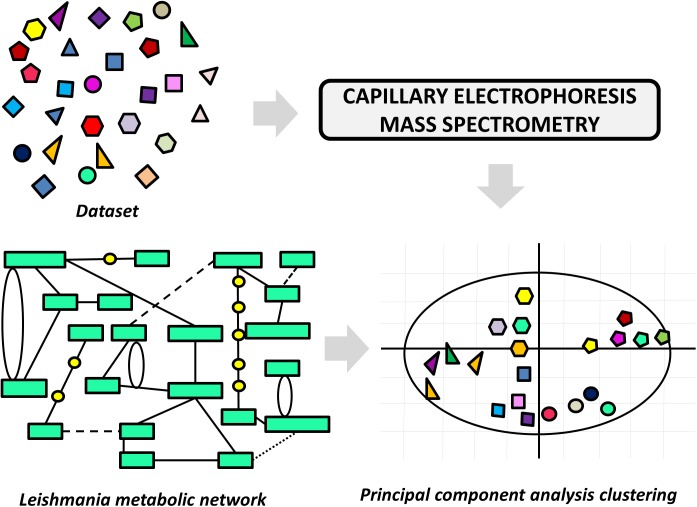
Ligand-based approach to classify compounds according to their mechanism of action. The effects of the dataset compounds on *Leishmania* metabolism were analyzed by capillary electrophoresis–mass spectrometry, and the data were used in a principal component analysis (PCA). The PCA was able to cluster compounds according to the perturbation they caused in the parasite’s metabolic network.

A variety of LBDD approaches have been recently reported in leishmaniasis drug discovery. These studies are frequently conducted in combination with experimental protocols and SBDD methods. The main goals include the use of QSAR and QSPR models to predict activity and ADMET parameters and the search for novel compounds via ligand-based virtual screening (LBVS). One of these studies reports an approach to pursuing novel compounds based on their effects on cell metabolism (Armitage et al., 2018). A collection of structurally diverse compounds, including those enclosed in the Leishmania box (a set of 592 compounds identified in HTS campaigns at GSK) (Peña et al., 2015) was evaluated in axenic *L. donovani* amastigotes, and the resulting metabolic changes were examined by capillary electrophoresis–mass spectrometry (Figure 6). Next, a principal component analysis (PCA) was applied to generate a model that assorts these compounds according to their putative mode of action. The authors demonstrated structural patterns involved in the modulation of different metabolic pathways and additionally, the role of physicochemical properties in the stimulation of individual biochemical routes. The study is very interesting, as it enables the classification of compound databases according to the most likely mechanism of action and biological outcomes. It also provides a way to run mechanistic studies of compounds that are known to be active against *Leishmania* species, thus offering a guide for downstream experimental profiling.

**FIGURE 7:**
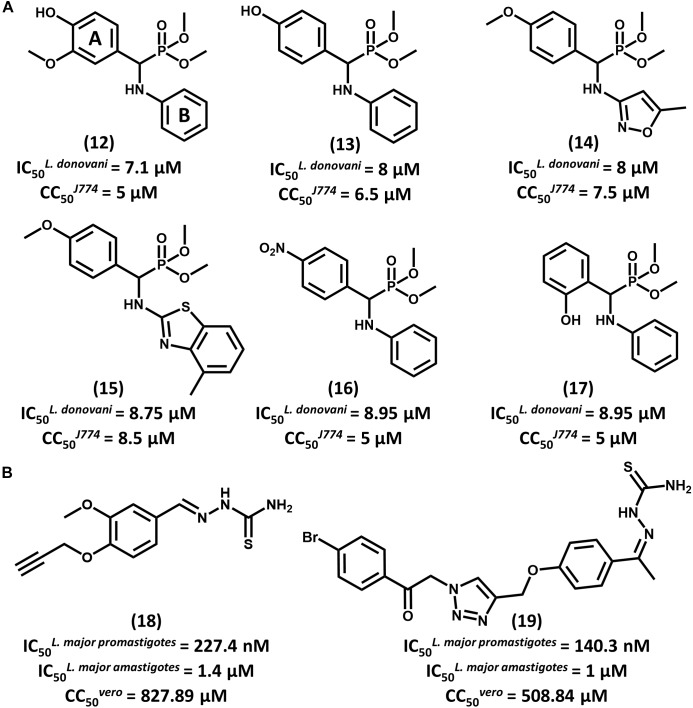
**(A)** A series of aminophosphonate derivatives as novel compounds featuring antiparasitic activity against *L. donovani* promastigotes. The QSAR models assigned rings A and B as the most relevant sites for molecular modification. **(B)** Triazole and thiosemicarbazone hybrids **18** and **19** showed promising activity against *L. major* promastigotes and amastigotes.

With the aid of QSAR modeling, Bhagat and coauthors described the synthesis and *in vitro* evaluation of 26 aminophosphonate derivatives (Bhagat et al., 2014). Six compounds (**12–17**, Figure 7A) displayed activity on *L. donovani* promastigotes in the low micromolar range (IC_50_ from 7.10 to 8.95 μM) and cytotoxicity on J774 macrophages comparable to that of amphotericin B. The authors took the gathered data for the whole compound series to build Comparative Molecular Field Analysis (CoMFA) models that have high predictive ability (*r*^2^_pred_ = 0.87) (Cramer et al., 1988). The models provided useful insights for future efforts on the optimization of this series. The CoMFA contour maps indicated that adding an electronegative group at the *para* position and a bulky electropositive substituent at the *meta* position in ring A would improve biological activity. Additionally, replacing ring B with substituted heterocyclic systems was stressed to be a worthwhile strategy for achieving more potent α-aminophosphonates as novel antileishmanial agents.

In a recent study, Temraz et al. (2018) reported the design of 1,2,3-triazole and thiosemicarbazone hybrids as novel antileishmanial compounds and the calculation of their ADMET profile. Out of the 17 evaluated molecules, most of them exhibited biological activity that is comparable or superior to that of the reference drug miltefosine. The most promising analogs, **18** and **19**, exhibited IC_50_ values of 227.4 and 140.3 nM, respectively, on *L. major* promastigotes (Figure 7B). On amastigotes, IC_50_ values of 1.4 and 1 μM were obtained for compounds **18** and **19**, respectively. The folate pathway was proposed as the target metabolic route, since folic acid reversed the antiparasitic activity. Toxicity data on VERO cells showed a selectivity profile that was superior to that of miltefosine (SI > 3000). Additionally, compounds **18** and **19** demonstrated no acute toxicity in mice at doses up to 125 mg/kg (oral) and 75 mg/kg (parenteral). Calculation of ADMET parameters demonstrated the drug-likeness of these compounds and their agreement with Lipinski’s rule of five. Considering the activity, selectivity, physicochemical and ADMET data, these triazole and thiosemicarbazone hybrids consist of promising lead compounds to be further investigated.

**FIGURE 8:**
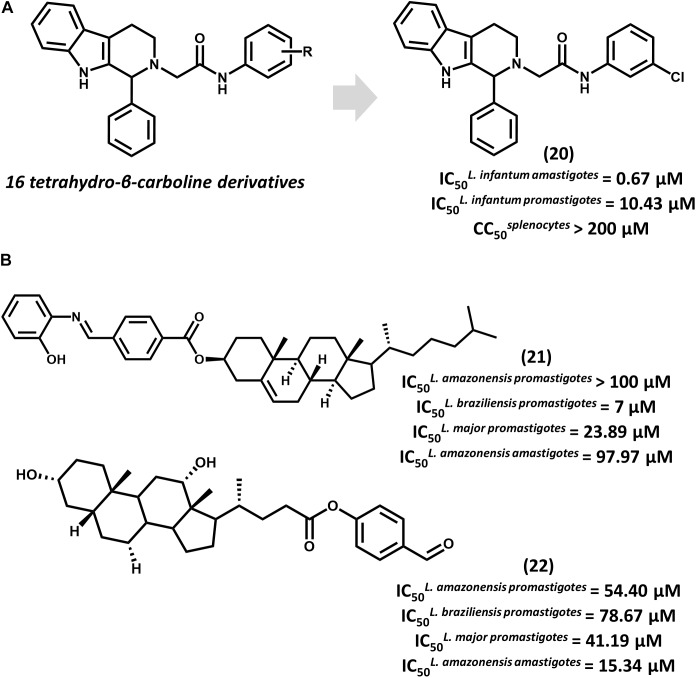
**(A)** The synthesis of a series of 16 tetrahydro-β-carboline derivatives led to compound **20** having promising *in vitro* activity against *L. infantum* promastigotes and amastigotes. **(B)** Cholesterol and deoxycholic acid derivatives **21** and **22** feature suitable activity against several *Leishmania* species.

Tetrahydro-β-carboline derivatives have recently been reported to have antileishmanial activity. In an investigation by Ashok et al. (2016) 16 analogs were designed, and most of them showed promising activity against *L. infantum* promastigotes (IC_50_ from 1.99 to 20.69 μM) and amastigotes (IC_50_ from 0.67 to 4.16 μM). Compound **20**, the most potent one (IC_50_ = 0.67 μM for amastigotes), showed activity comparable to that of amphotericin B (IC_50_ = 0.32 μM) and a selectivity index (SI) that is superior to 298 for the parasite over mammalian cells (Figure 8A). All compounds underwent QSPR studies for physicochemical profiling. Most analogs, including **20**, showed no violation of the Lipinski’s rule of five, demonstrating that they are likely to have good bioavailability. Given the gathered activity, selectivity and physicochemical data, this series consists of appropriate starting points for further investigation. Additional studies would be highly desirable for evaluating the *in vivo* reduction in parasite burden and hence, the potential of this series as novel drug candidates for leishmaniasis.

Steroid derivatives were described as novel antileishmanial agents in a recent report by da Trindade Granato et al. (2018). Out of the 16 synthesized analogs, cholesterol derivative **21** and some deoxycholic acid (DOA) derivatives proved active against *Leishmania* promastigotes (Figure 8B). Most DOAs were active against *L. amazonensis* intracellular amastigotes and displayed low toxic effects to macrophages. DOA **22** showed the best antiparasitic activity (IC_50_ = 15.34 μM) against amastigotes, which led to the investigation of its mechanism of action. Treatment of *L. amazonensis* with **22** led to the depolarization of the mitochondrial membrane potential and augmented reactive oxygen species (ROS) concentration, resulting in the arrest of the cell cycle. Estimation of ADMET properties revealed the suitability of **22** for oral administration. Additionally, the predictions indicated that this compound would have good blood-brain barrier permeation and would be susceptible to metabolic clearance by CYP3A4 enzymes. Further efforts to improve the *in vitro* activity of **22** and evaluate its *in vivo* efficacy would be worthwhile.

## Conclusion

A number of drug candidates are undergoing lead optimization studies and advanced *in vivo* preclinical profiling for leishmaniasis. Some of them could reach the clinical development phase, which have recently been filled by evaluations of different treatment regimens and combinations of previously approved drugs. Despite these advances and outcomes, it is prudent to adopt a conservative mindset given the long path that these compounds will have to take until potential approval and the high attrition rates that characterize pharmaceutical research. In this context, long-lasting efforts will be required to support state-of-the-art research programs that focus on the discovery of novel lead compounds for leishmaniasis. Such programs do exist today and have taken major advantage of the plentiful availability of data on *Leishmania*, as they move from trial-and-error to rational drug design. Current SBDD and LBDD campaigns have steadily contributed to rationalizing experimental data, thus providing effective insights into the design of optimized compounds. An important advance would be the validation of a higher number of molecular targets. Opportunely, some research centers have put intense efforts into this issue by developing large-scale chemical genomics and target deconvolution expertise. Regardless of the challenges ahead, chemoinformatics have been an important tool to prospect and profile promising compounds. This is corroborated by the findings discussed herein, which illustrate the rewarding integration of computational and experimental strategies in leishmaniasis drug R&D.

## Author Contributions

All authors listed have made a substantial, direct andintellectual contribution to the work, and approved it for publication.

## Conflict of Interest Statement

The authors declare that the research was conducted in the absence of any commercial or financial relationships that could be construed as a potential conflict of interest.

## Footnotes

1 http://www.who.int/leishmaniasis/en/

2 https://www.dndi.org/2013/media-centre/news-views-stories/news/first-early-stage-research-latin-america/

3 https://www.dndi.org/diseases-projects/portfolio/

## References

[B1] Abongomera C. Diro E. de Lima Pereira A. Buyze J. Stille K. Ahmed F. (2018). The initial effectiveness of liposomal amphotericin B (AmBisome) and miltefosine combination for treatment of visceral leishmaniasis in HIV co-infected patients in Ethiopia: a retrospective cohort study. *PLoS Negl. Trop. Dis.* 12:e0006527. 10.1371/journal.pntd.0006527 29799869 PMC5991765

[B2] Ansari M. Y. Dikhit M. R. Sahoo G. C. Ali V. Das P. (2017). Recent advancement and treatment of leishmaniasis based on pharmacoinformatics approach: current and future outlook. *Gene Rep.* 9 86–97. 10.1016/j.genrep.2017.09.003

[B3] Armitage E. G. Godzien J. Peña I. López-Gonzálvez Á. Angulo S. Gradillas A. (2018). Metabolic clustering analysis as a strategy for compound selection in the drug discovery pipeline for Leishmaniasis. *ACS Chem. Biol.* 13 1361–1369. 10.1021/acschembio.8b00204 29671577

[B4] Ashok P. Chander S. Tejería A. García-Calvo L. Balaña-Fouce R. Murugesan S. (2016). Synthesis and anti-leishmanial evaluation of 1-phenyl-2,3,4,9-tetrahydro-1H-β-carboline derivatives against *Leishmania infantum*. *Eur. J. Med. Chem.* 123 814–821. 10.1016/j.ejmech.2016.08.014 27541264

[B5] Berman H. M. Westbrook J. Feng Z. Gilliland G. Bhat T. N. Weissig H. (2000). The protein data bank. *Nucleic Acids Res.* 28 235–242. 10.1093/nar/28.1.235 10592235 PMC102472

[B6] Bhagat S. Shah P. Garg S. K. Mishra S. Kaur P. K. Singh S. (2014). α-Aminophosphonates as novel anti-leishmanial chemotypes: synthesis, biological evaluation, and CoMFA studies. *MedChemComm* 5 665–670. 10.1039/C3MD00388D

[B7] Brindisi M. Brogi S. Relitti N. Vallone A. Butini S. Gemma S. (2015). Structure-based discovery of the first non-covalent inhibitors of Leishmania major tryparedoxin peroxidase by high throughput docking. *Sci. Rep.* 5:9705. 10.1038/srep09705 25951439 PMC4423475

[B8] Casgrain P. A. Martel C. McMaster W. R. Mottram J. C. Olivier M. Descoteaux A. (2016). Cysteine peptidase B regulates *Leishmania mexicana* virulence through the modulation of GP63 expression. *PLoS Pathog.* 12:e1005658. 10.1371/journal.ppat.1005658 27191844 PMC4871588

[B9] Chen C. Y. (2013). A novel integrated framework and improved methodology of computer-aided drug design. *Curr. Top. Med. Chem.* 13 965–988. 10.2174/1568026611313090002 23651478

[B10] Copeland N. K. Aronson N. E. (2015). Leishmaniasis: treatment updates and clinical practice guidelines review. *Curr. Opin. Infect. Dis.* 28 426–437. 10.1097/QCO.0000000000000194 26312442

[B11] Cordeiro A. T. Feliciano P. R. Pinheiro M. P. Nonato M. C. (2012). Crystal structure of dihydroorotate dehydrogenase from Leishmania major. *Biochimie* 94 1739–1748. 10.1016/j.biochi.2012.04.003 22542640

[B12] Cramer R. D. Patterson D. E. Bunce J. D. (1988). Comparative molecular field analysis (CoMFA). 1. Effect of shape on binding of steroids to carrier proteins. *J. Am. Chem. Soc.* 110 5959–5967. 10.1021/ja00226a005 22148765

[B13] da Trindade Granato J. dos Santos J. A. Calixto S. L. da Silva N. P. da Silva Martins J., (2018). Novel steroid derivatives: synthesis, antileishmanial activity, mechanism of action, and in silico physicochemical and pharmacokinetics studies. *Biomed. Pharmacother.* 106 1082–1090. 10.1016/j.biopha.2018.07.056 30119174

[B14] De Luca L. Ferro S. Buemi M. R. Monforte A. M. Gitto R. Schirmeister T. (2018). Discovery of benzimidazole-based *Leishmania mexicana* cysteine protease CPB2.8ΔCTE inhibitors as potential therapeutics for leishmaniasis. *Chem. Biol. Drug Des.* 92 1585–1596. 10.1111/cbdd.13326 29729080

[B15] Dikhit M. R. Moharana K. C. Sahoo B. R. Sahoo G. C. Das P. (2014). LeishMicrosatDB: open source database of repeat sequences detected in six fully sequenced Leishmania genomes. *Database* 2014:bau078. 10.1093/database/bau078 25125444 PMC4132413

[B16] dos Santos R. N. Ferreira L. G. Andricopulo A. D. (2018). “Practices in molecular docking and structure-based virtual screening,” in *Computational Drug Discovery and Design. Methods in Molecular Biology*, eds Gore M. Jagtap U. (New York, NY: Humana Press), 31–50. 10.1007/978-1-4939-7756-7_3 29594766

[B17] Ferreira L. G. Dos Santos R. N. Oliva G. Andricopulo A. D. (2015). Molecular docking and structure-based drug design strategies. *Molecules* 20 13384–13421. 10.3390/molecules200713384 26205061 PMC6332083

[B18] Fiorillo A. Colotti G. Boffi A. Baiocco P. Ilari A. (2012). The crystal structures of the tryparedoxin-tryparedoxin peroxidase couple unveil the structural determinants of Leishmania detoxification pathway. *PLoS Negl. Trop. Dis.* 6:e1781. 10.1371/journal.pntd.0001781 22928053 PMC3424247

[B19] Folmer R. H. (2016). Integrating biophysics with HTS-driven drug discovery projects. *Drug Discov. Today* 21 491–498. 10.1016/j.drudis.2016.01.011 26826422

[B20] Gilbert I. H. (2013). Drug discovery for neglected diseases: molecular target-based and phenotypic approaches. *J. Med. Chem.* 56 7719–7726. 10.1021/jm400362b 24015767 PMC3954685

[B21] Hailu A. Dagne D. A. Boelaert M. (2016). “Leishmaniasis,” in *Neglected Tropical Diseases-Sub-Saharan Africa*, eds Gyapong J. Oatin B. (Berlin: Springer), 87–112. 10.1007/978-3-319-25471-5_5

[B22] Liu R. Li X. Lam K. S. (2017). Combinatorial chemistry in drug discovery. *Curr. Opin. Chem. Biol.* 38 117–126. 10.1016/j.cbpa.2017.03.017 28494316 PMC5645069

[B23] Logan-Klumpler F. J. De Silva N. Boehme U. Rogers M. B. Velarde G. McQuillan J. A. (2012). GeneDB–an annotation database for pathogens. *Nucleic Acids Res.* 40 D98–D108. 10.1093/nar/gkr1032 22116062 PMC3245030

[B24] Macalino S. Gosu V. Hong S. Choi S. (2015). Role of computer-aided drug design in modern drug discovery. *Arch. Pharm. Res.* 38 1686–1701. 10.1007/s12272-015-0640-5 26208641

[B25] Magariños M. P. Carmona S. J. Crowther G. J. Ralph S. A. Roos D. S. Shanmugam D. (2012). TDR Targets: a chemogenomics resource for neglected diseases. *Nucleic Acids Res.* 40 D1118–D1127. 10.1093/nar/gkr1053 22116064 PMC3245062

[B26] Mamidala R. Majumdar P. Jha K. K. Bathula C. Agarwal R. Chary M. T. (2016). Identification of *Leishmania donovani* Topoisomerase 1 inhibitors via intuitive scaffold hopping and bioisosteric modification of known Top 1 inhibitors. *Sci. Rep.* 6:26603. 10.1038/srep26603 27221589 PMC4879574

[B27] Marreiros B. C. Sena F. V. Sousa F. M. Oliveira A. S. Soares C. M. Batista A. P. (2017). Structural and Functional insights into the catalytic mechanism of the Type II NADH:quinone oxidoreductase family. *Sci. Rep.* 7:42303. 10.1038/srep42303 28181562 PMC5299459

[B28] Njogu P. M. Guantai E. M. Pavadai E. Chibale K. (2016). Computer-Aided drug discovery approaches against the tropical infectious diseases malaria, tuberculosis, Trypanosomiasis, and Leishmaniasis. *ACS Infect. Dis.* 2 8–31. 10.1021/acsinfecdis.5b00093 27622945

[B29] Ochoa R. Watowich S. J. Flórez A. Mesa C. V. Robledo S. M. Muskus C. (2016). Drug search for leishmaniasis: a virtual screening approach by grid computing. *J. Comput. Aided Mol. Des.* 30 541–552. 10.1007/s10822-016-9921-4 27438595

[B30] Ong H. B. Sienkiewicz N. Wyllie S. Fairlamb A. H. (2011). Dissecting the metabolic roles of pteridine reductase 1 in *Trypanosoma brucei* and Leishmania major. *J. Biol. Chem.* 286 10429–10438. 10.1074/jbc.M110.209593 21239486 PMC3060496

[B31] Patel P. Mandlik V. Singh S. (2016). LmSmdB: an integrated database for metabolic and gene regulatory network in Leishmania major and *Schistosoma mansoni*. *Genom. Data* 7 115–118. 10.1016/j.gdata.2015.12.012 26981382 PMC4778613

[B32] Peña I. Pilar Manzano M. Cantizani K. A. Alonso-Padilla J. Bardera A. I. (2015). New compound sets identified from high throughput phenotypic screening against three kinetoplastid parasites: an open resource. *Sci. Rep.* 5:8771. 10.1038/srep08771 25740547 PMC4350103

[B33] Pommier Y. Sun Y. Huang S. N. Nitiss J. L. (2016). Roles of eukaryotic topoisomerases in transcription, replication and genomic stability. *Nat. Rev. Mol. Cell. Biol.* 17 703–721. 10.1038/nrm.2016.111 27649880 PMC9248348

[B34] Ponder E. L. Freundlich J. S. Sarker M. Ekins S. (2014). Computational models for neglected diseases: gaps and opportunities. *Pharm. Res.* 2 271–277. 10.1007/s11095-013-1170-9 23990313

[B35] Preston S. Gasser R. B. (2018). Working towards new drugs against parasitic worms in a public-development partnership. *Trends Parasitol.* 34 4–6. 10.1016/j.pt.2017.07.005 28784352

[B36] Rashid U. Sultana R. Shaheen N. Hassan S. F. Yaqoob F. Ahmad M. J. (2016). Structure based medicinal chemistry-driven strategy to design substituted dihydropyrimidines as potential antileishmanial agents. *Eur. J. Med. Chem.* 115 230–244. 10.1016/j.ejmech.2016.03.022 27017551

[B37] Reguera R. M. Calvo-Álvarez E. Alvarez-Velilla R. Balaña-Fouce R. (2014). Target-based vs. phenotypic screenings in Leishmania drug discovery: a marriage of convenience or a dialogue of the deaf? *Int. J. Parasitol. Drugs Drug Resist.* 4 355–357. 10.1016/j.ijpddr.2014.05.001 25516847 PMC4266804

[B38] Rognan D. (2017). The impact of in silico screening in the discovery of novel and safer drug candidates. *Pharmacol. Ther.* 175 47–66. 10.1016/j.pharmthera.2017.02.034 28223231

[B39] Stevanović S. Perdih A. Senćanski M. Glišić S. Duarte M. Tomás A. M. (2018). In Silico Discovery of a Substituted 6-Methoxy-quinalidine with Leishmanicidal Activity in *Leishmania infantum*. *Molecules* 23:772. 10.3390/molecules23040772 29584709 PMC6017605

[B40] Sunyoto T. Potet J. Boelaert M. (2018). Why miltefosine-a life-saving drug for leishmaniasis-is unavailable to people who need it the most. *BMJ Glob. Health* 3:e000709. 10.1136/bmjgh-2018-000709 29736277 PMC5935166

[B41] Temraz M. G. Elzahhar P. A. El-Din A. Bekhit A. Bekhit A. A. Labib H. F. (2018). Anti-leishmanial click modifiable thiosemicarbazones: design, synthesis, biological evaluation and in silico studies. *Eur. J. Med. Chem.* 151 585–600. 10.1016/j.ejmech.2018.04.003 29656201

[B42] van Montfort R. L. M. Workman P. (2017). Structure-based drug design: aiming for a perfect fit. *Essays Biochem.* 61 431–437. 10.1042/EBC20170052 29118091 PMC5869280

[B43] Yousefinejad S. Hemmateenejad B. (2015). Chemometrics tools in QSAR/QSPR studies: a historical perspective. *Chemometr. Intell. Lab. Syst.* 149 177–204. 10.1016/j.chemolab.2015.06.016

